# Spontaneous Coronary Artery Dissection in a Patient with a Family History of Fatal Ascending Aortic Dissection: Case Report and Discussion of Diseases Causing Both Presentations

**DOI:** 10.1155/2019/7218480

**Published:** 2019-11-25

**Authors:** George Joy, Hany Eissa

**Affiliations:** ^1^Cardiology Department, St Bartholomew's Hospital, West Smithfield, London EC1A 7BE, UK; ^2^Cardiology Department, Queen Elizabeth Queen Mother Hospital, St Peter's Rd, Margate CT9 4AN, UK

## Abstract

**Background:**

Spontaneous coronary artery dissection (SCAD) is a rare cause of acute coronary syndrome (ACS). Aortic dissection and SCAD share common aetiologies such as a fibromuscular dysplasia (FMD), Marfan, Ehlers Danlos, and more rarely systemic lupus erythematosus and Loeys-Dietz; however, SCAD has never been known to have a familial association with aortic dissection.

**Case Summary:**

This case report describes a 48-year-old woman suffering from SCAD who had a mother who died from ascending aortic dissection in her 50s.

**Discussion:**

This is the first case report to our knowledge of a patient with SCAD with a first-degree relative with aortic dissection. Our case is interesting in that it shows that if predisposition to arterial dissection was inherited from mother to daughter, one of them suffered an extremely rare manifestation of their underlying disease. It also shows that a high index of suspicion is needed for SCAD in the presence of a patient with ACS and a family history of dissection elsewhere in the arterial tree.

## 1. Case Report

A 48-year-old woman was admitted to the emergency department with sudden onset severe chest tightness whilst doing yoga. This was associated with pins and needles in both arms, nausea, and abdominal discomfort. It lasted for 1 hour before it self-resolved and was not related to exertion. She reported being under increased emotional stress in the preceding month prior to presentation. She had no significant past medical history and was not on any regular medications.

She had a mother who died suddenly from an ascending aortic dissection in her 50s. Her mother was not hypertensive and suffered no symptoms or comorbidity suggestive of systemic illness.

**Figure 1:**
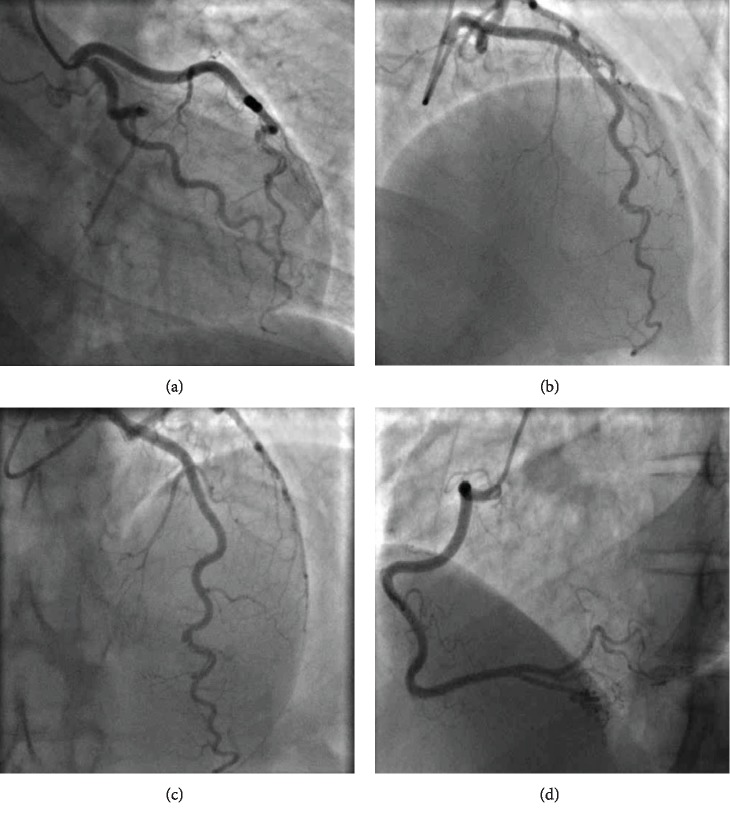
(a–c) Type 1 spontaneous coronary artery dissection (SCAD) (contrast dye staining of the arterial wall with multiple arterial lumens) in the distal LAD. Increased arterial tortuosity^∗^ in all three epicardial vessels including RCA (d). ^∗^Coronary artery tortuosity is highly prevalent in patients with spontaneous coronary artery dissection and is associated with recurrence.

The patient was hypotensive with a blood pressure of 90/60 mmHg and a heart rate of 80 bpm with no respiratory compromise or fever. Her troponin I (high-sensitivity assay) was 54 ng/dl, 79 ng/dl, and 27 ng/dl, respectively. Her electrocardiogram (ECG) showed sinus bradycardia with no ischaemic changes. A CT pulmonary angiogram was performed and excluded pulmonary embolus and showed no other cause for chest pain. An echocardiogram showed preserved biventricular function with no significant valvulopathy and normal ascending aorta dimensions. A coronary angiogram performed on day 2 of admission showed type 1 distal left anterior descending (LAD) coronary artery dissection with thrombolysis in antiplatelets and myocardial infarction (TIMI) 3 flow (Figure 1). She was initially managed with fondaparinux (factor Xa inhibitor) and dual antiplatelets with low-dose beta blocker long term once spontaneous coronary artery dissection (SCAD) was confirmed.

## 2. Discussion

Spontaneous coronary artery dissection (SCAD) is a cause of acute coronary syndrome (ACS) varying in severity from unstable angina to sudden cardiac death. This is the only case report to our knowledge of a patient with SCAD having a first-degree relative with aortic dissection. Twenty percent of patients with aortic dissection will have an underlying connective tissue disease [1]. We aim to discuss diseases that could link both presentations.

The incidence of patients with SCAD having fibromuscular dysplasia (FMD) has been reported as 74% [2]. Coronary FMD is rare and is characterised by dense intimal fibrous proliferation. Optical coherence tomography (OCT) may help establish the appearance of intima-media thickening; however, without this adjunctive imaging, FMD may only be diagnosed in extracoronary vessels in SCAD patients by CT or MRI [3]. A previous case report describing type B aortic dissection in a patient with FMD postulated this was more likely secondary to uncontrolled hypertension from renal FMD rather than primary arteriopathy in the aortic root [4]. Only three other case reports of aortic dissection from FMD have been described with one describing typical histopathologic features of FMD on the aortic root specimen postmortem [5]. Neither the patient nor her mother was hypertensive thereby making renal FMD less likely. Although overlaps with other connective tissue disease have been found, no definitive causative genes have been identified. The patient did not display any classical common clinical features of connective tissue disease, and she was unaware of the presence of these features in her mother [6]. Despite its rarity in aortic dissection, FMD appears to be the most likely culprit disease link between mother and daughter due to the lack of clinical features of other connective tissue disease outlined below.

Marfan syndrome (MFS) has an incidence of 1/10000 per year [7]. Isolated SCAD (i.e., not in association with aortic dissection) has been reported in Marfan syndrome and is extremely rare being described in five case reports to our knowledge occurring in young female patients with MFS. It is caused by cystic medial necrosis within the coronary artery leading to a predisposition to dissection [8]. Registry data has shown a prevalence of 4% of patients with ascending aortic dissection suffering from underlying Marfan disease [9]. Aortic dissection presents earlier in this group of patients within the 4^th^ and 6^th^ decade of life with a median age of 35. The aortic root is frequently more dilated prior to dissection (5.1 cm at the aortic root) causing an increased likelihood to suffer with aortic regurgitation prior to dissection [8]. Aortic dissection in MFS patients is more commonly not painful [1]. MFS is caused by autosomal dominant inheritance of FBN1 gene mutation encoding fibrillin 1 [7]. The patient did not meet diagnostic criteria for MFS including normal aortic dimensions, and therefore, MFS is unlikely to be the link between both patients.

Vascular Ehlers Danlos (vEDS) syndrome causing SCAD is extremely rare; we have identified seven case reports in the literature, including a patient in pregnancy with SCAD being the first presentation of vEDS [10–16]. Vascular EDS rarely affects the aorta, preferentially targeting first-order branch arteries. A review of 111 vEDS patients with vascular complications found only 10% suffering from aortic dissection [9]. vEDS is caused by autosomal dominant inheritance of COL3A1 mutation encoding for type 3 collagen and is commonly not diagnosed until vessel or hollow-organ rupture making it a suspect for the mother's presentation but would be an extremely rare manifestation in the daughter [17].

Some inflammatory conditions such as systemic lupus erythematosus (SLE) have been associated with SCAD in case reports, but this is highly uncommon; it is thought that 5% of SCAD have underlying inflammatory connective tissue disease [18]. Six case reports have described SCAD in association with SLE. A recent case report attributed SCAD as a first presentation of SLE, however without clinical criteria for SLE and positive ANA and anti-SMA [19]. Systematic review has identified 40 previous cases published of SLE with aortic aneurysm and or dissection [20]. Inflammatory causes are unlikely in our patient as blood tests a few months before her diagnosis were negative for ANA, rheumatoid factor, and ESR.

Loeys-Dietz syndrome is a rare connective tissue disorder that is caused by autosomal dominant inheritance of TGFBR-1 mutation that causes defects in elastogenesis leading to arterial tortuosity, aneurysm, and rupture. This affects any segment of the aorta, but has rarely been described in case reports to affect the coronary artery. Other associated features include cleft palate/uvula, hypertelorism, craniosynostosis, and other congenital heart defects such as ASD or bicuspid aortic valve [21]. The absence of these features makes Loeys-Dietz unlikely in our patient or her mother.

## 3. Conclusion

Our case shows a presentation of SCAD with an unusual family history. The main learning points from this case are that a high index of suspicion of SCAD is needed in a young patient presenting with ACS and that connective tissue disease and extracoronary arterial screening needs to be considered in these situations. There is no obvious disease that links both presentations although we propose FMD as the most likely culprit. It is clear from literature review that if the predisposition to dissection was inherited from mother to daughter, one of them suffered a rare manifestation of their underlying disease. SCAD is a life-changing event for the patient due to the complications of myocardial infarction and the threat of lifelong recurrence.
